# Growth and Nutritional Responses of Bean and Soybean Genotypes to Elevated CO_2_ in a Controlled Environment

**DOI:** 10.3390/plants8110465

**Published:** 2019-10-30

**Authors:** José Soares, Teresa Deuchande, Luísa M.P. Valente, Manuela Pintado, Marta W. Vasconcelos

**Affiliations:** 1 CBQF—Centro de Biotecnologia e Química Fina—Laboratório Associado, Escola Superior de Biotecnologia, Universidade Católica Portuguesa, Rua Diogo Botelho 1327, 4169-005 Porto, Portugal; jsoares@porto.ucp.pt (J.S.); tdeuchande@porto.ucp.pt (T.D.); mpintado@porto.ucp.pt (M.P.); 2 CIIMAR – Centro Interdisciplinar de Investigação Marinha e Ambiental, Universidade do Porto, Avenida General Norton de Matos, 4450-208 Matosinhos, Portugal; lvalente@icbas.up.pt; 3 ICBAS, Instituto de Ciências Biomédicas de Abel Salazar, Universidade do Porto, Rua de Jorge Viterbo Ferreira, 228, 4050-313 Porto, Portugal

**Keywords:** bean, elevated CO_2_, controlled environment, mineral concentrations, seed yield, soybean

## Abstract

In the current situation of a constant increase in the atmospheric CO_2_ concentration, there is a potential risk of decreased nutritional value and food crop quality. Therefore, selecting strong-responsive varieties to elevated CO_2_ (eCO_2_) conditions in terms of yield and nutritional quality is an important decision for improving crop productivity under future CO_2_ conditions. Using bean and soybean varieties of contrasting responses to eCO2 and different origins, we assessed the effects of eCO_2_ (800 ppm) in a controlled environment on the yield performance and the concentration of protein, fat, and mineral elements in seeds. The range of seed yield responses to eCO_2_ was −11.0 to 32.7% (average change of 5%) in beans and −23.8 to 39.6% (average change of 7.1%) in soybeans. There was a significant correlation between seed yield enhancement and aboveground biomass, seed number, and pod number per plant. At maturity, eCO_2_ increased seed protein concentration in beans, while it did not affect soybean. Lipid concentration was not affected by eCO2 in either legume species. Compared with ambient CO_2_ (aCO_2_), the concentrations of manganese (Mn), iron (Fe), and potassium (K) decreased significantly, magnesium (Mg) increased, while zinc (Zn), phosphorus (P), and calcium (Ca) were not changed under eCO_2_ in bean seeds. However, in soybean, Mn and K concentrations decreased significantly, Ca increased, and Zn, Fe, P, and Mg concentrations were not significantly affected by eCO_2_ conditions. Our results suggest that intraspecific variation in seed yield improvement and reduced sensitivity to mineral losses might be suitable parameters for breeders to begin selecting lines that maximize yield and nutrition under eCO_2_.

## 1. Introduction

With the worldwide population predicted to increase to almost 9.5 billion by 2050, a larger portion of the essential nutrients for humans will be provided by plant-based sources [1,2]. The regular consumption of plant proteins, including that of grain legumes, can reduce the risk of diet-related diseases like obesity, diabetes, cardiovascular problems, hypertension, stroke, and cancers that have been increasing in previous decades [3]. Consequently, legumes could be considered an important part of the human diet, as they are a good source of minerals, proteins, vitamins, and bioactive compounds [4]. Among the grain legumes cultivated, dry beans and soybeans are regarded as important crops, and the European Union highlighted the importance of increasing their production to reduce external requirements, and decrease possible negative impacts associated with intensive cereal production [5], thus improving farming sustainability. An overview from 2000 to 2017 reported an increase from 500 Kt to 1.1 Mt, and from 1.9 Mt to 10.7 Mt in dry bean and soybean production in Europe, respectively [6]. However, among European countries, Portugal has a diminutive production of beans equivalent to 1.7 Kt, and in the case of soybean, the production is practically non-existent.

Plant growth is dependent on some resources, including water, mineral nutrients, light, and CO_2_ [7]. The effects of elevated CO_2_ (eCO_2_) on plant responses is an important topic and has been the subject of scientific research. Nevertheless, there is a lack of information about the genotypic variation of eCO_2_ responses on yield and grain quality parameters, particularly in legume species. The atmospheric CO_2_ concentration has raised almost 12%, from nearly 370 ppm in 2000 to almost 413 ppm in 2019 [8], surpassing anything that plants had to deal with millions of years ago. In this manner, eCO_2_ is typically considered as either a positive or a negligible driver of photosynthesis, growth, and yield, mainly on C3 plants [9]. However, differences in the range of yield stimulation are usually detected [10], and a significant intraspecific variation in responses to eCO_2_ has been found in rice [11,12,13], cowpea [14], wheat [15], common bean [16], and soybean [17,18]. These variations in eCO_2_ responsiveness suggest that selecting and breeding genotypes that respond positively to eCO_2_ may ensure sustained productivity and improve food security in an upcoming high CO_2_ world [19].

Simultaneously, this trend of increasing ambient CO_2_ (aCO_2_) levels, which are projected to reach 550 ppm by the middle of this century, is possibly threatening human nutrition, even if further actions are taken to reduce emissions (IPCC, 2014). Consequently, the concentration of various grain mineral elements is influenced to a great extent by eCO_2_ conditions [20]. Myers et al. [2], in a meta-analysis, evaluated the response of several crops grown at aCO_2_ and eCO_2_ in free-air CO_2_ enrichment (FACE) conditions. Elevated CO_2_ was associated with significant decreases in the concentration of zinc (Zn) and iron (Fe) in the edible parts of rice, wheat, field peas, and soybeans. In another study, a decrease in the overall mineral concentrations (a change of −8%) was observed in several C3 crops, reflecting foliar and edible tissues, FACE and non-FACE studies [21]. Other studies also reported decreased nutritional value in edible parts of C3 crops due to eCO_2_ conditions [22,23,24]. Furthermore, eCO_2_ was associated with lower protein concentration in the edible parts of rice, wheat, barley, potato, field peas [2], and vegetables [25], but not in soybean, combining FACE and growth chamber data [2]. Further characteristics of seed quality are also maintained at eCO_2_ in legumes, such as grain crude fat on beans, mung bean, and soybean [26,27,28]. So, there is still a need to explore genotypic variability, among legume species, that reveal an improved seed yield and nutritional responsiveness to eCO_2_ levels.

In the present study, we focused on the intraspecific variation of two legume species on yield responses under eCO_2_ in a controlled environment, simultaneously assessing aspects associated with the nutritional quality.

## 2. Results

### 2.1. Genotypic Variation of Yield Responses to eCO_2_

**Figure 1:**
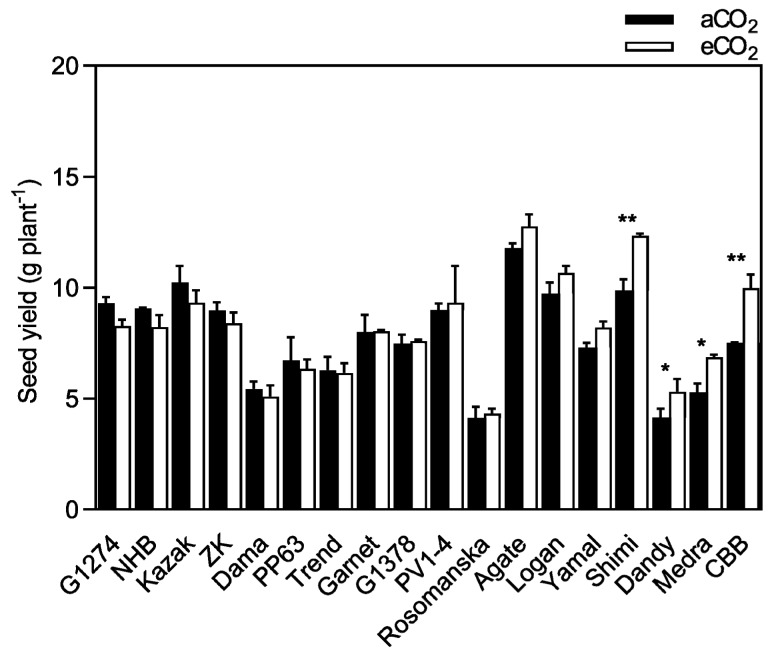
Seed yield of bean grown under ambient CO_2_ (aCO_2_) (400 ppm) and elevated CO_2_ (eCO_2_) (800 ppm). Data are means ± SE (*n* = 10 plants). From left to right, varieties are classified in order of increasing seed yield responsiveness to eCO_2_. ** *p* < 0.01; * *p* < 0.05 significance level.

A significant increase in seed yield due to eCO_2_ was observed in beans, with a mean response of 5.0% (*p* < 0.05), as demonstrated in Figure 1 and Table 1. The rank of seed yield improvement was greatest for Chocolate Brown Bean (CBB, 32.7%), followed by Medra (30.3%), Dandy (28.0%), and Shimi (25.0%) varieties. These were considered strong-responsive varieties under eCO_2_ conditions (see Section 4.1). Besides, no significant differences were observed among the remaining varieties due to eCO_2_. Agate had the highest seed yield at both CO_2_ concentrations. The extent of seed yield improvement due to eCO_2_ differed significantly among the varieties (*p* < 0.0001), with a significant CO_2_ x variety interaction (*p* < 0.05), as demonstrated in Table 1.

The aboveground biomass (sum of the weights of stems, pod shells, seeds) at maturity was significantly increased by eCO_2_ (*p* < 0.05), and there was a significant intraspecific variation associated with eCO_2_ (*p* < 0.0001) without a significant CO_2_ x variety interaction (*p* > 0.05). The biomass response was strongly correlated with yield increase to eCO_2_ (r = 0.747, *p* < 0.01). On the other hand, the harvest index, which was expressed as the ratio of seed yield to aboveground biomass, was not changed by eCO_2_ (*p* > 0.05). Further, there was no significant correlation between harvest index and yield enhancement due to eCO_2_ conditions (Table 1).

The relative increase in height in response to eCO_2_ was 4.8% (*p* < 0.05; Table 1), and the magnitude of this increase differed significantly between varieties (*p* < 0.0001), without a significant CO_2_ x variety interaction (*p* > 0.05). Further, we observed a strong correlation between yield response to eCO_2_ and relative increase in height (r = 0.593, *p* < 0.01).

Of the yield components, exposure to eCO_2_ resulted in a significant stimulation on the number of seeds per pod (mean CO_2_ effect of 7.5%, *p* < 0.01; Table 1), and the magnitude of this increase differed significantly among the varieties (*p* < 0.0001), without a CO_2_ x variety interaction (*p* > 0.05). Moreover, a correlation between increased seed yield and an increased number of seeds per pod was not observed (*p* > 0.05).

Elevated CO_2_ resulted in seed mass reduction by −13.1% (*p* < 0.0001), but there was no significant correlation between seed mass reduction and yield improvement (*p* > 0.05). No significant differences were observed in the number of pods (mean CO_2_ effect of 2.9%, *p* > 0.05) and in the number of seeds per plant (mean CO_2_ effect of 3.8%, *p* > 0.05) due to eCO_2_. However, a significant intraspecific variability was observed (*p* < 0.0001) with a significant CO_2_ x variety interaction (*p* < 0.05) for both yield components. There was a strong positive correlation between the number of pods (r = 0.736, *p* < 0.01) and the number of seeds per plant (r = 0.838, *p* < 0.01) with seed yield enhancement (Table 1).

**Figure 2:**
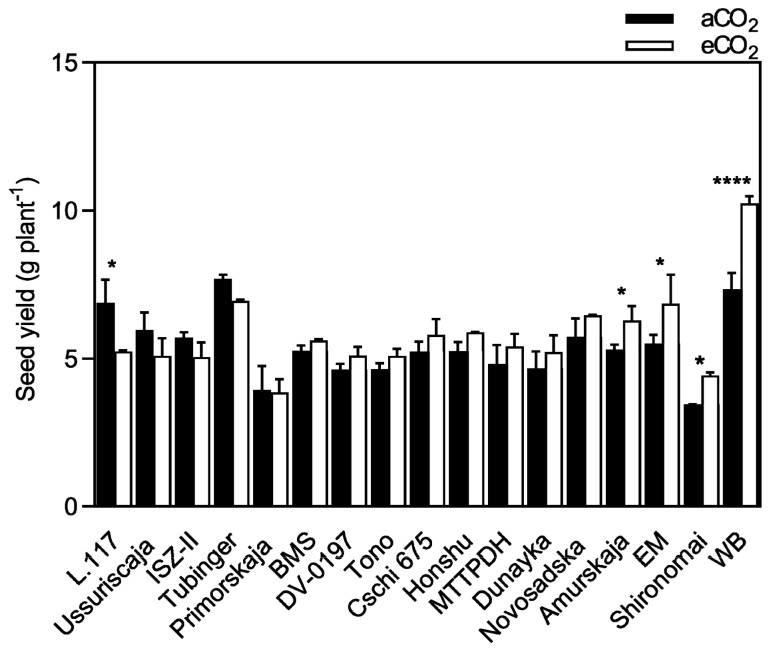
Seed yield of soybean grown under aCO_2_ (400 ppm) and eCO_2_ (800 ppm). Data are means ± SE (*n* = 10 plants). From left to right, varieties are classified in order of increasing seed yield responsiveness to eCO_2_. **** *p* < 0.0001; * *p* < 0.05 significance level.

Concerning soybean, CO_2_ enrichment significantly stimulated seed yield by an average of 7.1% (*p* < 0.05; Figure 2 and Table 2). This magnitude of seed yield enhancement differed significantly among the varieties (*p* < 0.0001), and there was a significant CO_2_ x variety interaction (*p* < 0.01). The largest seed yield increase at eCO_2_ was observed in Wisconsin Black (WB, 39.6%), Shironomai (28.5%), and Early Mandarin (24.5%), which were considered strong-responsive varieties, followed by Amurskaja (18.4%). No significant differences in seed yield were observed among the remaining cultivars, except for L.117 (*p* < 0.05), which showed a significant decrease in seed yield under eCO_2_. At aCO_2_, WB with Tubinger had the highest seed yield, which was consistent at eCO_2_, whereas WB significantly surpassed all other varieties (Figure 2).

The aboveground biomass was significantly increased by 6.9% due to eCO_2_ (*p* < 0.05, Table 2), and there was a significant difference among the varieties (*p* < 0.0001), without a CO_2_ x variety interaction (*p* > 0.05). This increase in biomass was significantly correlated with seed yield enhancement at eCO_2._ (r = 0.625, *p* < 0.01). The harvest index was not affected by eCO_2_ (*p* > 0.05), with a significant intraspecific variation (*p* < 0.0001). Further, there was no significant correlation between harvest index and yield enhancement due to eCO_2_ conditions (Table 2).

On the other hand, a significant increase in height due to eCO_2_ was observed, with an average response of about 4%. The magnitude of this enhancement due to eCO_2_ differed significantly among the varieties (*p* < 0.0001), with a significant CO_2_ x variety interaction (*p* < 0.0001, Table 2).

Of the yield components, eCO_2_ had significant effects on pod number per plant (mean CO_2_ effect of 7.2%, *p* < 0.01), seed number per plant (mean CO_2_ effect of 5.5%, *p* < 0.05), seed number per pod (mean CO_2_ effect of 5.9%, *p* < 0.05), and 100-seed weight (mean CO_2_ effect of −12.3%, *p* < 0.0001). The extent of all reproductive parameters differed significantly among the varieties (*p* < 0.05), with a significant CO_2_ x variety interaction (*p* < 0.05), except on the number of pods per plant (*p* > 0.05, Table 2). Moreover, there was a strong and positive correlation between seed yield improvement and pod number per plant (r = 0.784, *p* < 0.01), seed number per plant (r = 0.600, *p* < 0.05), and seed number per pod (r = 0.665, *p* < 0.01), as described in Table 2.

### 2.2. Variation of Grain Nutritional Composition Due to eCO_2_

**Figure 3:**
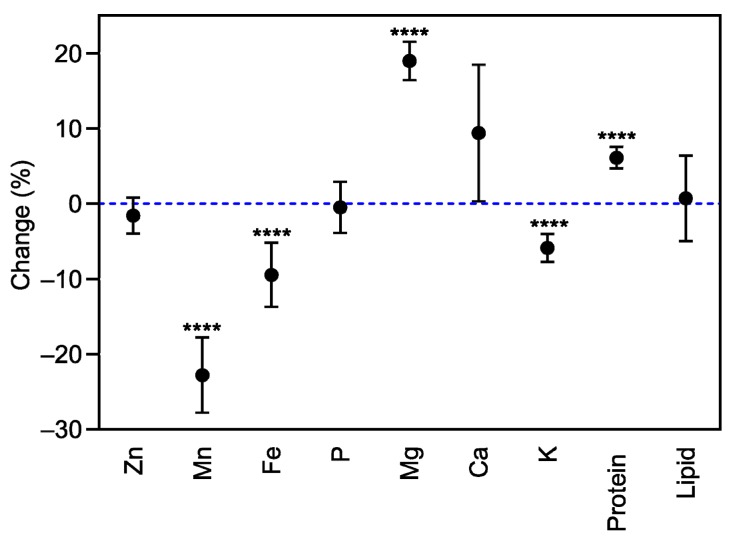
Mean response change (%) of the seed mineral, protein, and lipid concentrations of 18 bean varieties grown under aCO_2_ (400 ppm) and eCO_2_ (800 ppm). **** *p* < 0.0001 significance level.

**Figure 4:**
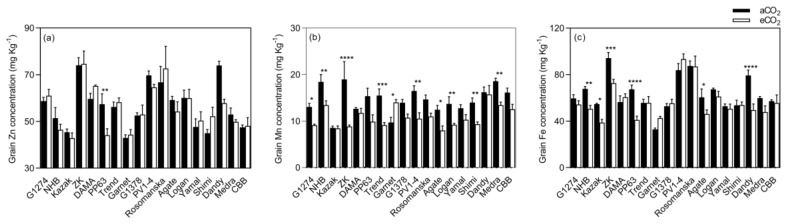
Grain micronutrient (**a**–**c**) concentrations of bean grown under aCO_2_ (400 ppm) and eCO_2_ (800 ppm). Each bar represents the mean ± SE (*n* = 10 plants). * *p* < 0.05; ** *p* < 0.01; *** *p* < 0.001; **** *p* < 0.0001 significance level.

Elevated CO_2_ did not influence Zn, P, or Ca concentrations in bean seeds at maturity (*p* > 0.05, Figure 3). However, the concentrations of the other minerals (viz. Mn, Fe, Mg, and K) responded differently to eCO_2_. Under eCO_2_, the Mn concentration was significantly decreased by 25.2% (*p* < 0.0001). The decrease was significant in 9 out of 18 varieties, whereas it increased in Garnet (*p* < 0.05), and in Kazak, Dama, PP63, G1378, Rosomanska, Yamal, Dandy, and CBB, no changes were observed at eCO_2_ (Figure 4).

The Fe concentration was decreased by 39.1%, 37.6%, 29.0%, 25.4%, 23.7%, and 22.9% (*p* < 0.001) in PP63, Dandy, Kazak, North Holland Bruine (NHB), Agate, and Zlaty Knot, respectively (Figure 4).

**Figure 5:**
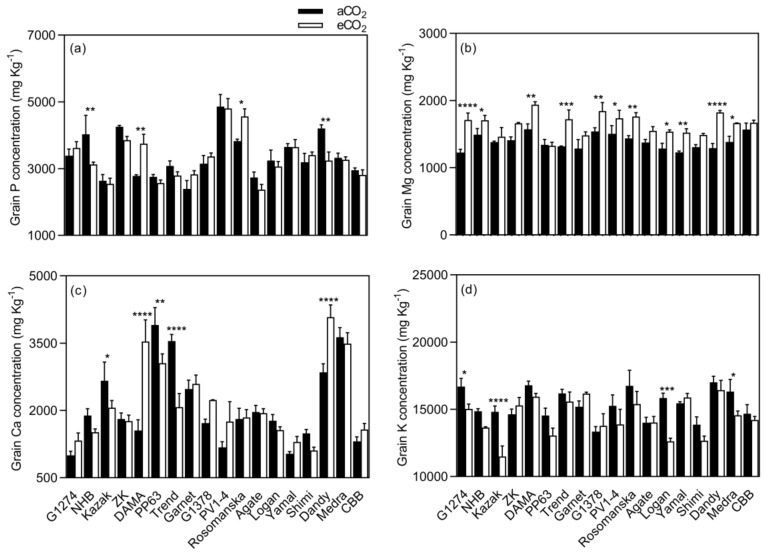
Grain macronutrient (**a**–**d**) concentrations of bean grown under aCO_2_ (400 ppm) and eCO_2_ (800 ppm). Each bar represents the mean ± SE (*n* = 10 plants). * *p* < 0.05; ** *p* < 0.01; *** *p* < 0.001; **** *p* < 0.0001 significance level.

Grain Mg concentration increased under eCO_2_ for G1274, NHB, Dama, Trend, G1378, PV1-4, Rosomanska, Logan, Yamal, Dandy, and Medra and remained unchanged in the rest of the varieties (Figure 5). Significant changes in K concentration were observed in G1274, Kazak Logan, and Medra (Figure 5), which showed a decrease in grain K concentration (mean CO_2_ effect of −6.0%, *p* < 0.05, Figure 3), while no changes were demonstrated in the remaining varieties. The extent of change in all grain mineral concentrations in response to eCO_2_ varied between varieties (Table 3, *p* < 0.01), implying a significant CO_2_ x cultivar interaction (*p* < 0.01).

**Figure 6:**
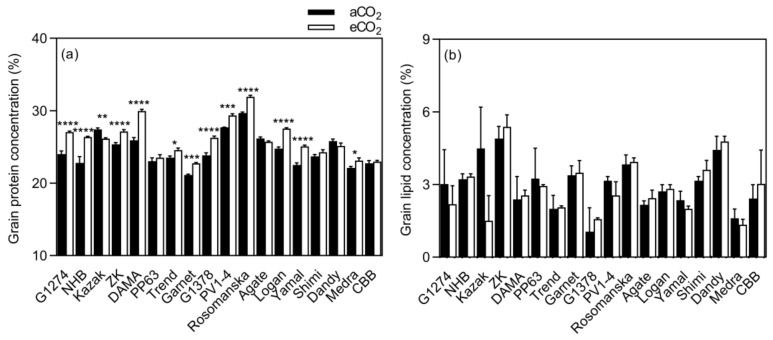
Influence of eCO_2_ on bean seed protein and lipid concentrations. Each bar represents the mean ± SE (*n* = 10 plants). * *p* < 0.05; ** *p* < 0.01; *** *p* < 0.001; **** *p* < 0.0001 significance level.

Exposure to eCO_2_ significantly increased protein concentration when compared to aCO_2_ (mean CO_2_ effect of 6.0%, *p* < 0.0001, Figure 3). The increase was significant in 12 out of 18 varieties, while decreased in Kazak (*p* < 0.05), and in Agate, CBB, Dandy, PP63, and Shimi, the concentration remained unchanged (Figure 6). A significant effect of CO_2_ × variety interaction on protein concentration was observed (*p* < 0.0001, Table 3). Elevated CO_2_ had no influence on fat concentration in all bean varieties at maturity when compared to aCO_2_ (Figure 3).

**Figure 7:**
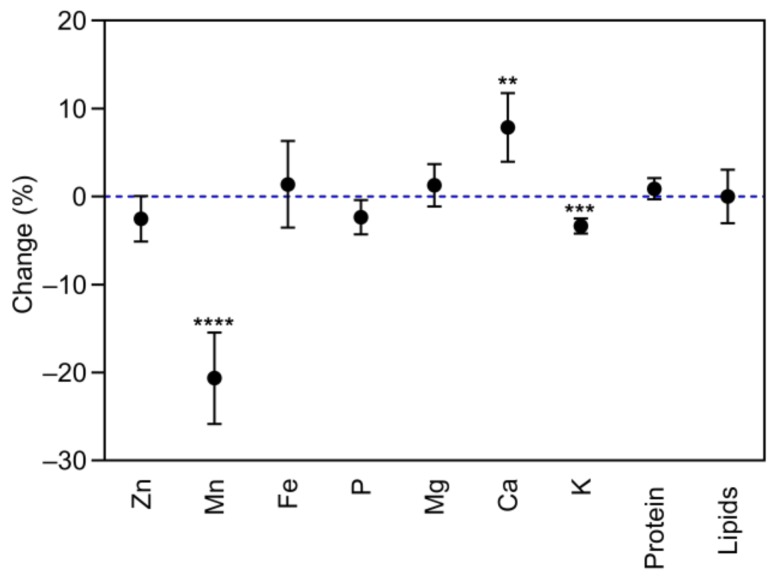
Mean response change (%) of the seed mineral, protein, and lipid concentrations of 17 soybean varieties grown under aCO_2_ (400 ppm) and eCO_2_ (800 ppm). ** *p* < 0.01; *** *p* < 0.001; **** *p* < 0.0001 significance level.

**Figure 8:**
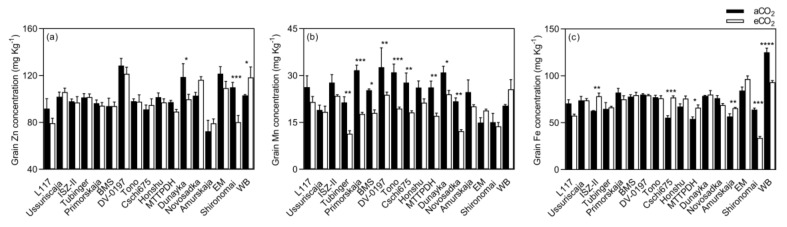
Grain micronutrient (**a**–**c**) concentrations of soybean grown under aCO_2_ (400 ppm) and eCO_2_ (800 ppm). Each bar represents the mean ± SE (*n* = 10 plants). * *p* < 0.05; ** *p* < 0.01; *** *p* < 0.001; **** *p* < 0.0001 significance level.

In soybean, eCO_2_ did not influence Zn, Fe, P, or Mg concentrations in seeds (*p* > 0.05, Figure 7). On the other hand, eCO_2_ significantly decreased grain Mn concentration by 23.2% (*p* < 0.0001). The concentration of this element decreased in Tubinger, Primorskaja, Bai Mao Shuang, DV-0197, Tono, Cschi675, Man-tsan-tszinxPhin-di-Huan (MTTPDH), Dunayka, and Novosadska, and no significant differences were observed in the remaining varieties (Figure 8).

**Figure 9:**
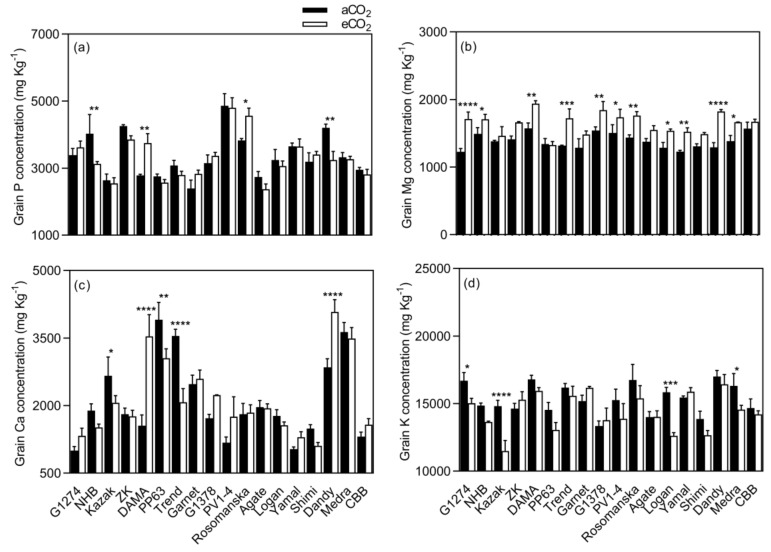
Grain macronutrient (**a**–**d**) concentrations of soybean grown under aCO_2_ (400 ppm) and eCO_2_ (800 ppm). Each bar represents the mean ± SE (*n* = 10 plants). * *p* < 0.05; *** *p* < 0.001 significance level.

**Figure 10:**
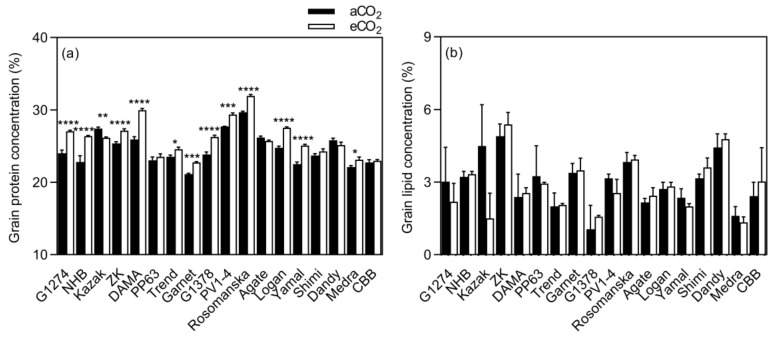
Influence of eCO_2_ on soybean seed protein and lipid concentrations. (**a**) soybean seed protein and (**b**) lipid concentrations. Each bar represents the mean ± SE (*n* = 10 plants). * *p* < 0.05; ** *p* < 0.01 significance level; *** *p* < 0.001; **** *p* < 0.0001 significance level.

Elevated CO_2_ significantly increased grain Ca concentration by 36.3%, 34.9%, 25.3%, and 24.3% in ISZ-II, Amurskaja, Ussuriscaja, Tubinger, respectively, decreased by 21.5% in Primorskaja, and was not affected in the remaining varieties (Figure 9). Furthermore, eCO_2_ decreased K concentration by 3.5% (*p* < 0.001) when compared to aCO_2_. The response of grain mineral concentrations to eCO_2_ varied between varieties (Table 4, *p* < 0.01), implying a significant CO_2_ x cultivar interaction (*p* < 0.01), except for P concentration. Also, eCO_2_ had no influence on the grain protein and lipid concentrations (*p* > 0.05, Figure 7) in soybean. However, the extent of change in grain protein and lipid concentrations in response to eCO_2_ varied between varieties (*p* < 0.001, Figure 10 and Table 4).

## 3. Discussion

Strong-responsive genotypes to eCO_2_ may be crucial and might support significant yield increases in a future eCO_2_ environment. The increased performance must encompass not only productivity at the whole-plant level, but must also nutritional resilience to future climate conditions.

The current study demonstrated that under eCO_2_, seed yield differed substantially among the varieties tested (*p* < 0.0001), ranging from −11.0 to 32.7% in bean, and from −23.8 to 39.6% in soybean (Figure 1 and Figure 2), suggesting a considerable genetic background for genomic improvement. It was also previously demonstrated that yield responses to increasing CO_2_ varied greatly, among varieties and between species, ranging from −10 to 80% for soybean [18,19,29,30] and from −11 to 39% for common bean [16,31]. Nevertheless, eCO_2_ increased the seed yield but failed to improve the harvest index; however, decreases in harvest index due to CO_2_ enrichment can occur in soybean [32]. Similar results have been reported in lupin [33], where exposure to eCO_2_ did not decrease the harvest index, because the effect of CO_2_ was mainly an increase in biomass and, consequently, an increase in the number of pods that reached maturity and the number of pods with filled seeds. Herein, the seed yield increase was 5.0% (bean) and 7.1% (soybean), which is relatively lower than other reports [16,18,19,29]. This failure of seed yield increase is possibly associated with the physical restriction to root growth, since the volume of the containers for root growth was <2 L. It is widely accepted that the pot size significantly affects seed yield responses to eCO_2_, since plants grown in larger pots (>9 L) have greater stimulation compared to those grown in small pots [32]. Also, the CO_2_-induced reduction in seed mass, which may be a consequence of the restriction of nutrient production, mobilization, and translocation to the seeds during seed filling, is probably associated with the physical restriction of root growth.

However, the driving force in the yield-enhancing strategy was linked to the response of biomass to eCO_2_ and, subsequently, to the number of pods and seeds production, and these were probably useful indicators of the intraspecific variation (Table 1 and Table 2). This is in agreement with Kumagai et al. [19], who reported the growth of soybean in a greenhouse at eCO_2_. The authors showed that cultivars with the strongest responsiveness of biomass to eCO_2_ produced more pods and greater seed yield. Bunce [16] also demonstrated seed yield improvement under eCO_2_, among common bean varieties, and a high correlation with stimulation of pod and seed numbers. Therefore, it was proposed that a genotype with higher sink formation due to eCO_2_ would be a promising candidate for higher yield responses to eCO_2_ [17].

However, it is important to understand whether the characteristics that lead to higher responsiveness to eCO_2_ are also manifested under aCO_2_ for the development of effective plant breeding strategies [16]. In the current study, the highest yielding variety at aCO_2_ was the highest yielding variety at eCO_2_ in both species. Therefore, Agate (bean) and WB (soybean) have a higher yield at both concentrations. This suggests that varieties best adapted to current CO_2_ levels may also have the characteristics best adapted to future CO_2_ concentrations, providing good genetic support for future studies.

The impact of eCO_2_ on the grain nutritional quality has also been studied, since CO_2_ enrichment can lead to a decrease in plant nutritional status, and pose a potential challenge to human health [20]. Elevated CO_2_ significantly reduced the grain nutritional value in terms of Mn, Fe, and K in bean, and Mn and K in soybean (Figure 3 and Figure 7). Similar results for Mn and K have been reported by Loladze [21] in a wide range of C3 crops, reflecting foliar and edible tissues, FACE and non-FACE studies, and by Myers et al. [2] in field peas. The reduction in grain Fe content due to eCO_2_ has also been reported in rice, wheat, barley, peas, and soybeans [2,20].

Furthermore, exposure to eCO_2_ increased Mg and Ca concentrations in bean and soybean, respectively. Similar results were obtained by Li et al. [20] in soybean seeds at the fresh edible and mature stages. On the other hand, grain Zn and P concentrations were not influenced by eCO_2_ in either species. Dong et al. [25] in vegetables and Li et al. [20] in soybean also found that P concentration was not affected by eCO_2_.

The mechanisms responsible for reducing the concentration of nutrients associated with eCO_2_ have not yet been fully clarified. Many studies attribute this to the carbohydrate dilution effect, where increasing plant biomass under eCO_2_ conditions dilutes the rest of the grain components [20,34,35,36]. Our findings were contradictory, with carbohydrate dilution functioning alone since we found that mineral changes within the same species are distinct from each other, suggesting that the mechanism is more complex than carbohydrate dilution alone. For example, in bean (Figure 3), the decrease in Mn concentration was significantly different from the decrease in Fe concentration or K concentration, and the increase in Mg concentration. It also seems that the mechanisms causing these changes function distinctly in different species. Consequently, we found Mg concentration to be significantly increased in bean (*p* < 0.0001), whereas it was not changed in soybean grains (*p* > 0.05, Figure 7). Therefore, eCO_2_ has both positive and negative effects on the nutritional quality of legume seeds. Inhibition of photorespiration and malate production [37], carbohydrate dilution, and decreased mass flow due to reduced transpiration may all be relevant to explain this phenomenon of decreased grain nutritional value under eCO_2_ conditions [38,39].

We also examined the effects of eCO_2_ on mineral concentrations as a function of variety. Both crops showed significant differences across varieties among all minerals studied (Table 3 and Table 4). Such changes among varieties suggest a basis for breeding varieties whose reduced nutrient levels are less responsive to eCO_2_.

Legumes are a major source of proteins and oil, particularly soybean, containing essential free amino acids and fatty acids [20]. Concerning grain protein concentration, it was demonstrated that eCO_2_ increased grain protein in bean (*p* < 0.0001) and had no influence in soybean seeds (*p* > 0.05, Figure 3 and Figure 4), with significant differences among varieties (Table 3 and Table 4). These findings that protein concentration was less affected are also associated with the competence of leguminous crops to counteract the stimulation of photosynthetic C gain at eCO_2_, with better nitrogen fixation for preserving tissue C:N ratios [40]. Our results are in agreement with those of Jablonski et al. [41], who, in a meta-analysis of several crops and wild species, found that seed protein was not affected by high CO_2_ concentrations in legumes, but declined significantly in most non-legumes. Similarly, Taub and Wang [42] indicated that eCO_2_ did not affect soybean seed protein concentration. Myers et al. [2] also found that eCO_2_ was associated with lower protein concentration in wheat and rice grains, and a non-significant effect of eCO_2_ was demonstrated in soybeans or C4 crops grown under FACE conditions.

Few studies dealing with the effects of eCO_2_ on plant lipid metabolism have been carried out. In this study, it was demonstrated that eCO_2_ had no effect on lipid concentration in bean and soybean grains (*p* > 0.05, Figure 3 and Figure 7). Similar results were reported in *Arabidopsis thaliana* [43], wheat [22], and soybean grains [20] at the fresh edible stages and grown at eCO_2_.

It was previously demonstrated that eCO_2_ decreased the concentrations of Fe and Zn in grains of most C3 plants [20,22,25,44], and usually, C3 crops other than legumes also have lower concentrations of protein [2]. These dietary deficiencies are considered a global public health problem, as it is estimated that two billion people worldwide are affected by these nutritional deficiencies [2]. Therefore, strong-responsive cultivars (i.e., CBB, Medra, and Shimi in bean, and EM in soybean) in terms of seed yield enhancement and that maintain or even increase Fe, Zn, and grain protein concentrations at eCO_2_ might be considered as promising varieties for future studies.

## 4. Materials and Methods 

### 4.1. Plant Material

In this study, we used bean (*Phaseolus vulgaris* L.) and soybean (*Glycine max* L.) varieties, that were obtained either from CIAT (Cali, Colombia) or from USDA-ARS via Germplasm Resources Information Network (Washington, USA). Varieties of both species were chosen based on a preliminary experiment (aCO_2_, 400 ppm and eCO_2_, 600 ppm) conducted under FACE conditions at Campus Klein (Altendorf, Germany) to find out the performance under eCO_2_. The seed yield response (strong-responsive with >25% vs. weak-responsive with <25% of yield increase) at eCO_2_ was based on average seed yield responses under eCO_2_ and reported by [16,18,19,29,30,45,46]. In the selected varieties, the growth and yield performance at eCO_2_ were assessed in a controlled environment (Table 5).

### 4.2. Growth Conditions

The experiment was conducted from January to May in 2017, at the Grow to Green facility (Castelo Branco, Portugal). Seeds were sown on phenolic foam plugs, and seven days after sowing (DAS), seedlings were transplanted to the growth chamber. Plants were grown in a thin nutrient film solution in polyvinyl chloride-coated gullies and placed with 0.20 m in between. Irrigation was performed through 10 min ON/15 min OFF during light period; and 10 min ON/30 min OFF during night period. Plants grew with a photoperiod of 16/8 h (day/night) at an average light intensity expressed as photosynthetic photon flux density of 350 μmol m^−2^ s^−1^ at canopy level. Light conditions were provided by LED lamps with peak emissions of 650, 540, and 460 nm for Red/White/Blue (80:6:14) light, with ratio representing the contribution of red, white, and blue light to total intensity. The temperature was kept at 25/20 °C (day/night) and relative humidity at 75%. Electric conductivity and pH in the nutrient solution were registered by sensors and automatically readjusted to 0.60 mS m^−1^ and 5.5, respectively. The composition of the nutrient solution for hydroponic growth included: 1.2 mM KNO_3_, 0.8 mM Ca(NO_3_)_2_, 0.3 mM MgSO_4_.7H_2_O, 0.2 mM NH_4_H_2_PO_4_, 25 µM CaCl_2_, 25 µM H_3_BO_3_, 0.5 µM MnSO_4_, 2 µM ZnSO_4_.H_2_O, 0.5 µM CuSO_4_.H_2_O, 0.5 µM MoO_3_, 0.1 µM NiSO_4_, and 20 µM FeEDDHA. The experiment was conducted at eCO_2_ (800 ppm) and aCO_2_ (400 ppm) concentrations until maturity in two independent growth chambers. There were two replicates, with five plants per replicate, in each treatment arranged in a randomized block design.

### 4.3. Growth and Yield Measurements

For all genotypes, SPAD values were determined at 54 DAS at the pod formation stage. Following senescence of the foliage and discoloration of the pods between 9–10 weeks, irrigation was discontinued, and plants allowed to dry in situ. Pods were hand harvested at maturity between 79–99 DAS depending on the variety. At maturity, aboveground dry weight (sum of the weights of stems, pods shells, and seeds), plant’s height, number of pods per plant, number of seeds per plant, and the average weight of 100 seeds were performed for all varieties in both treatments. Seed yield per plant was obtained from ten plants (*n* = 2 replicates) and adjusted to a 15% moisture content.

### 4.4. Nutritional Analysis

Seeds from independent plants (*n* = 4 replicates) were collected and analyzed for minerals, protein N, and total lipid concentration. Mineral analysis determination was performed as described by Santos et al. [47]. The minerals analyzed were Zn, Fe, manganese (Mn), phosphorous (P), magnesium (Mg), calcium (Ca), and potassium (K). Briefly, 200 mg of the seed material was mixed with 5 mL of 65% HNO_3_ (*v*/*v*) and 1 mL of H_2_O_2_ 30% (*v*/*v*) in a Teflon reaction vessel and heated in a SpeedwaveTM MWS-3+ (Berghof, Germany) microwave system. Digestion procedure was conducted in five steps, consisting of different temperature and time sets: 130 °C/10 min, 160 °C/15 min, 170 °C/12 min, 100 °C/7 min, and 100 °C/3 min. The resulting clear solutions of the digestion procedure were then brought to 50 mL with ultrapure water for further analysis. Mineral concentration determination was performed using the ICP-OES Optima 7000 DV (PerkinElmer, USA) with radial configuration.

Seeds were analyzed for crude protein concentration (N x 5.28 and N x 5.5 in bean and soybean, respectively) using a Leco nitrogen analyzer (Model FP-528, Leco Corporation, St. Joseph, USA), and crude fat concentration was measured by petroleum ether extraction (40–60 °C) using a Soxhlet fat extraction system (Gerhardt, Germany). All chemical analyses followed AOAC [48] methods.

### 4.5. Statistical Analysis

To test for significant differences between CO_2_ treatments and among varieties, and for significant interactions, plant data were analyzed as a completely randomized design using a two-way ANOVA. The correlations among seed yield and agronomic traits were performed using Pearson’s product-moment correlation (r). All statistical analyses were performed with version 25.0 of the SPSS statistics software.

## 5. Conclusions

In summary, our results indicate that consistent and significant variation in the response of seed yield to eCO_2_ under controlled conditions does exist among legume species, and that the response of pod and seed numbers are suitable for predicting their responsiveness to future eCO_2_. Moreover, Mn and K concentrations were significantly decreased by eCO_2_ in both species. The protein concentration in bean seeds was significantly increased. Lipid concentrations were not influenced by eCO_2_ in the present study Thus, it is important to develop specially designed programs to increase seed yield while avoiding or reducing some of the important nutritional losses that may arise under eCO_2_ conditions.

## Figures and Tables

**Table 1:** Growth and reproductive characteristics at maturity of 18 bean varieties grown at ambient (400 ppm) and elevated (800 ppm) CO_2_, and correlations (Pearson’s r) and their statistical significance for the relationship between the relative increase in bean seed yield due to eCO_2_ (value at eCO_2_/value at aCO_2_) and values of other parameters measured under the same conditions. * *p* < 0.05; ** *p* < 0.01; *** *p* < 0.001; **** *p* < 0.0001. C x V, CO_2_ x variety interaction; ns, not significant.

Parameter	Mean CO_2_ Effect	CO_2_	Variety	C x V	Correlation	
Aboveground dry weight, g plt^−1^	5.8%	*	****	ns	0.747	*
Height, cm plt^−1^	4.8%	*	****	ns	0.593	**
Seed yield, g plant^−1^	5.0%	*	****	*	‒	‒
Harvest index, g g^−1^	−0.2%	ns	**	ns	0.096	ns
No. of pods, plt^−1^	2.9%	ns	****	*	0.736	*
No. of seeds, plt^−1^	3.8%	ns	****	***	0.838	**
No. of seeds, pod^−1^	7.5%	**	****	ns	0.314	ns
100-seed weight, g	−13.1%	****	****	*	−0.108	ns

**Table 2:** Growth and reproductive characteristics at maturity of 17 soybean varieties grown at either ambient (400 ppm) and elevated (800 ppm) CO_2_, and correlations (Pearson’s r) and their statistical significance for the relationship between the relative increase in bean seed yield due to eCO_2_ (value at eCO_2_/value at aCO_2_) and values of other parameters measured under the same conditions. * *p* < 0.05; ** *p* < 0.01; **** *p* < 0.0001.

Parameter	Mean CO_2_ Effect	CO_2_	Variety	C x V	Correlation	
Aboveground dry weight, g plt^−1^	6.9%	*	****	ns	0.625	**
Height, cm plt^−1^	3.6%	*	****	****	0.119	ns
Seed yield, g plt^−1^	7.1%	*	****	**	‒	
Harvest index, g g^−1^	−1.0%	ns	****	ns	0.396	ns
No. of pods, plt^−1^	7.2%	**	****	ns	0.784	**
No. of seeds, plt^−1^	5.5%	*	****	****	0.600	*
No. of seeds, pod^−1^	5.9%	*	*	*	0.665	**
100-seed weight, g	−12.3%	****	****	**	−0.280	ns

**Table 3:** Significance levels of main effects and interactions of CO_2_ and varieties on bean grain nutrient, protein, and lipid concentrations at maturity. ns, not significant; ** *p* < 0.01; *** *p* < 0.001; **** *p* < 0.0001.

Seed Element	CO_2_	Variety	C x V
Zn	ns	****	ns
Mn	****	****	***
Fe	****	****	****
P	ns	****	**
Mg	****	****	ns
Ca	ns	**	****
K	****	****	**
Protein	****	****	****
Lipid	ns	**	ns

**Table 4:** Significance levels of main effects and interactions of CO_2_ and varieties on soybean grain nutrient, protein, and lipid concentrations at maturity. ns, not significant; ** *p* < 0.01; *** *p* < 0.001; **** *p* < 0.0001.

Seed Element	CO_2_	Variety	C x V
Zn	ns	****	**
Mn	****	****	**
Fe	ns	****	****
P	ns	****	ns
Mg	ns	****	**
Ca	**	***	**
K	***	****	**
Protein	ns	****	**
Lipid	ns	***	ns

**Table 5:** List of bean (*n* = 18) and soybean (*n* = 17) varieties grown at aCO_2_ (400 ppm) and eCO_2_ (800 ppm). Performance at eCO_2_ was obtained from a preliminary FACE experiment to find out the strong-responsive (>25% yield increase) and weak-responsive (<25% yield increase) varieties against eCO_2_.

Crop	Acession Number	Growth Habit	Common Name	Origin	Performance at eCO_2_
Bean ^a^	PI 203929	D	G1274	Mexico	Strong-responsive
Bean ^a^	PI 458586	D or I	NHB	Netherlands	Strong-responsive
Bean ^b^	PI 169920	D	Kazak	Turkey	Weak-responsive
Bean ^a^	PI 324691	D	ZK	Hungary	Weak-responsive
Bean ^a^	W6 9628	I	Dama	Czechoslovakia	Weak-responsive
Bean ^a^	W6 12428	NS	PP 63	Bulgaria	Strong-responsive
Bean ^a^	PI 550128	I	Trend	Netherlands	Weak-responsive
Bean ^a^	PI 550038	NS	Garnet	United States	Weak-responsive
Bean ^b^	PI 212027	D	G1378	Iran	Weak-responsive
Bean ^a^	PI 598287	I	PV1-4	Japan	Weak-responsive
Bean ^a^	PI 368715	D or I	Rosomanska	Macedonia	Strong-responsive
Bean ^a^	PI 550035	D	Agate	United States	Weak-responsive
Bean ^b^	PI 149484	D	Logan	United States	Weak-responsive
Bean ^a^	PI 136687	D	Yamal	Canada	Weak-responsive
Bean ^a^	PI 165933	D	Shimi	India	Weak-responsive
Bean ^a^	PI 550037	D	Dandy	United States	Strong-responsive
Bean ^b^	G 8853	D	Medra	Germany	Strong-responsive
Bean^a^	PI 477023	D or I	CBB	Netherlands	Strong-responsive
Soybean ^a^	PI 361085 A	I	L.117	Romania	Strong-responsive
Soybean ^a^	PI 437413	I	Ussurijscaja	Russia	Weak-responsive
Soybean ^a^	PI 424194	D	ISZ-II	Hungary	Weak-responsive
Soybean ^a^	PI 445823	I	Tubinger	Germany	Weak-responsive
Soybean ^a^	PI 378676 A	I	Primorskaja	Russia	Strong-responsive
Soybean ^a^	PI 561302 A	I	BMS	China	Weak-responsive
Soybean ^a^	PI 437101	I	DV-0197	Russia	Weak-responsive
Soybean ^a^	PI 319537 A	I	Tono	China	Strong-responsive
Soybean ^a^	PI 437224	I	CSchi 675	Moldova	Strong-responsive
Soybean ^a^	PI 319534 A	I	Honshu	China	Strong-responsive
Soybean ^a^	PI 437676 A	I	MTTPDH	China	Weak-responsive
Soybean ^a^	PI 445829 A	I	Dunayka	Romania	Strong-responsive
Soybean ^a^	PI 361097 A	I	Novosadska	Serbia	Strong-responsive
Soybean ^a^	PI 360952	I	Amurskaja	Russia	Weak-responsive
Soybean ^a^	PI 417554	I	EM	Poland	Strong-responsive
Soybean ^a^	PI 538409	D	Shironomai	Japan	Strong-responsive
Soybean ^a^	PI 153271	I	WB	Belgium	Strong-responsive

^a^ Obtained from GRIN; ^b^ obtained from CIAT; D, determinate; I, indeterminate; NS, not specified; NHB, North Holland Bruine; ZK, Zlaty Knot; CBB, Chocolate Brown Bean; BMS, Bai mao Shuang¸ MTTPDH, Man-tsan-tszinxPhin-di-Huan; EM, Early Mandarin; WB, Wisconsin Black.
